# Leveraging neuro-inspired AI accelerator for high-speed computing in 6G networks

**DOI:** 10.3389/fncom.2024.1345644

**Published:** 2024-02-21

**Authors:** Chunxiao Lin, Muhammad Farhan Azmine, Yibin Liang, Yang Yi

**Affiliations:** Bradley Department of Electrical and Computing Engineering, Virginia Tech, Blacksburg, VA, United States

**Keywords:** echo state network, 6G, massive MIMO, OFDM, AI, FPGA

## Abstract

The field of wireless communication is currently being pushed to new boundaries with the emergence of 6G technology. This advanced technology requires substantially increased data rates and processing speeds while simultaneously requiring energy-efficient solutions for real-world practicality. In this work, we apply a neuroscience-inspired machine learning model called echo state network (ESN) to the critical task of symbol detection in massive MIMO-OFDM systems, a key technology for 6G networks. Our work encompasses the design of a hardware-accelerated reservoir neuron architecture to speed up the ESN-based symbol detector. The design is then validated through a proof of concept on the Xilinx Virtex-7 FPGA board in real-world scenarios. The experiment results show the great performance and scalability of our symbol detector design across a range of MIMO configurations, compared with traditional MIMO symbol detection methods like linear minimum mean square error. Our findings also confirm the performance and feasibility of our entire system, reflected in low bit error rates, low resource utilization, and high throughput.

## 1 Introduction

Since the 3GPP fifth-generation (5G) standard was proposed in 2015, it has brought great evolution to many areas, such as mobile applications, autonomous vehicles, the Internet of Things, smart cities, etc. (Sah et al., 2019). Enhanced by many key technologies such as millimeter wave (mmWave) frequencies, multiple-input multiple-output (MIMO), 5G is capable of providing higher data rates, high user density, and lower latency. However, the theoretical peak capability of 5G can be predicted from the 3GPP technical specifications. For new applications such as holographic communication, virtual reality, and remote robotic surgery, the sixth-generation (6G) wireless network, is expected to provide ultra-high bandwidth and ultra-low latency (Saad et al., 2019).

Antennas in 6G devices can be compact in size due to ultra-high mmWave frequency. Therefore, the use of large arrays of antennas becomes one of the characteristics of 6G networks for the purpose of performance improvement. Novel multiple-antenna technologies such as massive MIMO (mMIMO), extremely large MIMO (XL-MIMO), and cell-free mMIMO (CF-mMIMO) are proposed in this situation.

Massive MIMO, which builds on existing MIMO technology, increases the number of antennas at base stations to tens or hundreds, leading to a significant improvement in throughput and efficiency (Rusek et al., 2012). To make the most of this advancement, both network systems and mobile devices must be designed in a more complex way to coordinate MIMO operations, which brings new technical challenges. In particular, symbol detection is one of the key challenges to tackle in massive MIMO. Conventional methods based on channel state information (CSI) estimation can handle the MIMO system with a limited number of antennas. However, obtaining accurate CSI for an extremely large number of antennas is a hard task with high computational complexity. In this case, AI-based symbol detection methods become good candidates for massive MIMO systems.

Among different AI models, the echo state network (ESN) shows a good potential to handle the challenge of symbol detection. ESN is part of the broader concept of reservoir computing, which is inspired by the way how biological brains process information. As a variation of recurrent neural networks, ESN is able to model the complicated and dynamic channel in massive MIMO. Compared to traditional neural networks, ESN shows better computational efficiency due to its fixed reservoir layer. Furthermore, ESN has a low requirement for the size of the training dataset. This makes ESN a better option for massive MIMO symbol detection tasks since there are limited data symbols available for data training in high-speed wireless communication. The dataset we use for ML model training will be discussed later in Section 2.4, where only a small part of the transmitted data symbols can be used in the training.

Another benefit brought by ESN to the symbol detection task is converting the task running in serial into a single-instruction-multi-data (SIMD) task, introducing the possibility of being accelerated by computation-efficient hardware like field programmable gate array (FPGA). FPGA accelerator, with high parallelism, can implement SIMD tasks in a much more cost-effective way. Computation-specific circuit designs in FPGA platforms can greatly increase processing speed and reduce power consumption. Therefore, we can improve the performance of the massive MIMO system by designing an FPGA-accelerated ESN symbol detector.

### 1.1 Relevant prior art

Although massive MIMO is a hot research topic, the research on the detection algorithms has been started decades ago. For small-scale MIMO, simple detection algorithms like a matched filter (MF) can show good performance (Marzetta, 2010). When it comes to practical medium-size massive MIMO systems, these simple algorithms would produce unacceptable results (Wu et al., 2014). Therefore, other linear schemes, such as the zero-forcing (ZF) and minimum mean square error (MMSE) detectors (Tuchler et al., 2002a,b), are widely used nowadays. These methods are based on the estimation of the wireless channel and further detection on the estimated channel. And in Neumann et al. (2015) and Xie et al. (2016), different improvements for channel estimation were proposed, like the semi-blind method to suppress pilot contamination interference (Neumann et al., 2015), and low-rank methods to reduce effective channel dimensions (Xie et al., 2016). In terms of another challenge of computational complexity, AI-based methods have shown greater performance.

AI techniques are widely used to replace conventional methods in wireless communication (Wang et al., 2017; Qin et al., 2019; Hoydis et al., 2021; Liu et al., 2021). And more AI-based methods are applied to massive MIMO tasks, such as channel estimation (He et al., 2018), CSI compression (Wen et al., 2018), and precoding matrix design (Sohrabi et al., 2021).

Echo state networks, known as the brain-inspired RNN with low training overhead and low computation complexity, are also explored a lot in the wireless domain, mainly focusing on the symbol detection task in OFDM symbol detection tasks. Mosleh et al. (2017) has proven that ESN-based methods perform well in MIMO-OFDM symbol detection. Further improvements for the ESN symbol detector were proposed by Zhou et al. (2020a,b).

The introduction of ESN successfully solves the problem of MIMO-OFDM symbol detection in terms of computational efficiency. However, FPGA-based ESN designs further accelerate the symbol detection task with much lower resource utilization and power consumption. Gan et al. (2021) and Lin et al. (2022) explored the design of ESN reservoir neurons with different architectures in SISO-OFDM symbol detection. These two accelerators decrease the resource utilization of the ESN with validations on the FPGA board, showing the potential of FPGA acceleration in the MIMO-OFDM system. In our work, we further explore the FPGA acceleration in both the MIMO and massive MIMO systems.

### 1.2 Contribution and outline

In this research, significant contributions are made to AI acceleration for 6G networks. The first contribution is the application of the ESN method to massive MIMO-OFDM symbol detection. Additionally, we introduce a cost-efficient ESN neuron architecture using advanced digital signal processing (DSP) to accelerate the ESN symbol detector. Furthermore, we verified the performance and efficiency with sufficient simulations for different MIMO configurations. Finally, the architecture is validated through a proof-of-concept implementation on an FPGA board, demonstrating its practice efficiency.

The remainder of this paper is structured as follows. Section II introduces the massive MIMO-OFDM system and the ESN-based symbol detector. The architecture design for ESN acceleration is then described in Section III. Then in Section IV, we display the simulation results of the ESN-based symbol detector for different MIMO configurations. Section V is dedicated to the proof of concept of our AI accelerator on a jointed software-defined radio (SDR) / FPGA testbed. The paper concludes with Section VI, where we summarize the key takeaways in this work and acknowledge the limitations of our study.

## 2 Background

### 2.1 Massive MIMO-OFDM

**Figure 1:**
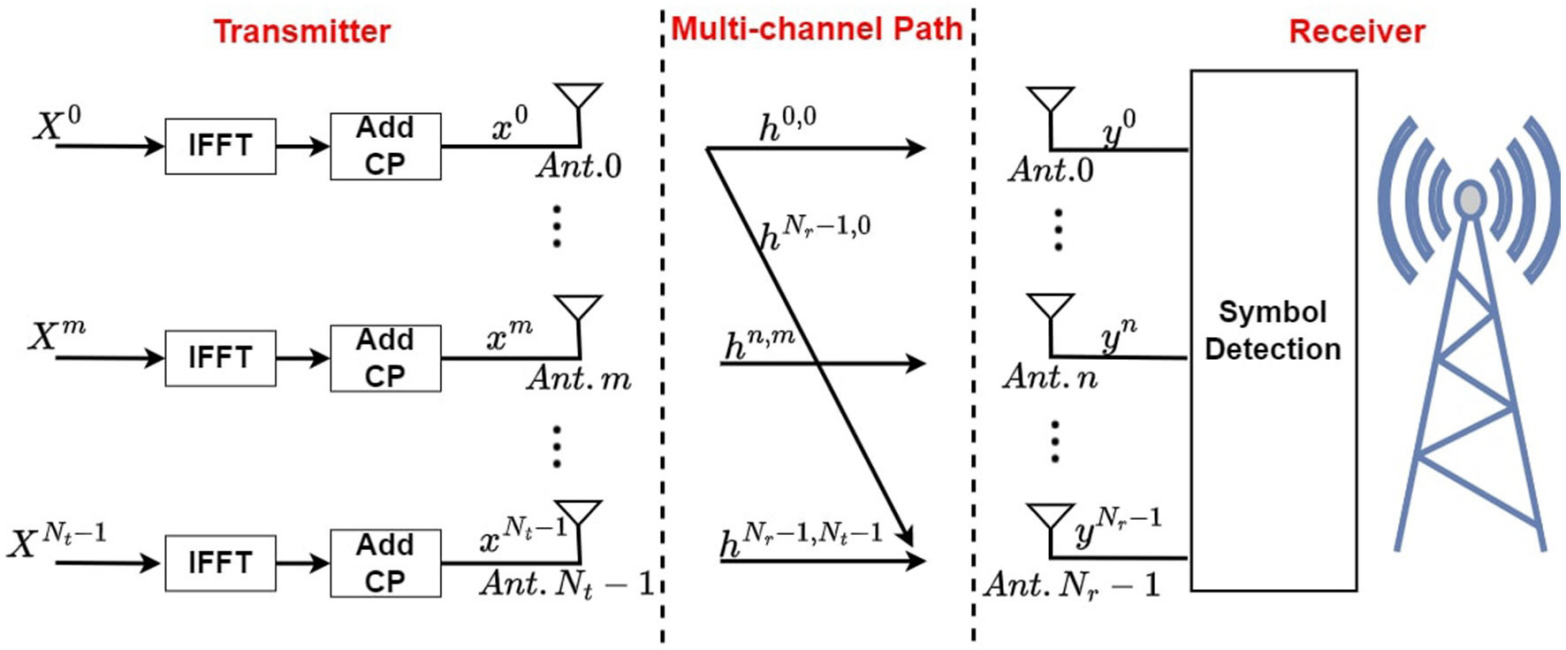
Massive MIMO-OFDM system for uplink transmission.

The massive MIMO-OFDM (mMIMO) architecture is demonstrated in Figure 1, where an uplink transmission is displayed, allowing data and signals to be transmitted from the user device to the base station (BS) using an uplink channel. In this mMIMO system, there are *N*_*t*_ antennas at the transmitter side, sending *N*_*t*_ independent data streams to the wireless channel. Prior to the transmission of signals, an inverse fast Fourier transform (IFFT) is performed to convert each signal from the frequency domain to the time domain. And the last *N*_*cp*_ symbols of each signal are copied and inserted to the beginning as cyclic prefix (CP). At the receiver side, *N*_*r*_ receiver antennas are used for data stream reception. The *i*th frequency domain OFDM symbol for *t*th data stream are denoted as Equation 1:


(1) $$\begin{array}{l} {\tilde{X}}_{i}^{t}≜{\left[{\tilde{X}}_{i}^{t}(0),\cdots ,{\tilde{X}}_{i}^{t}(k),\cdots ,{\tilde{X}}_{i}^{t}(N_{sc}-1)\right]}^{T} \end{array}$$

where ·^*T*^ denotes matrix transposing operation, and *N*_*sc*_ stands for the total number of sub-carriers in each OFDM symbol. For subcarrier *k*, ${\tilde{X}}_{i}^{t}\left(k\right)$ is considered as the QAM symbol after modulation.

For case of all the data streams, the *i*th frequency-domain QAM symbols at *k*th sub-carrier are weighted using one precoding matrix $Q\left(k\right)\in {\mathbb{C}}^{N_{t}\times N_{t}}$ before doing OFDM modulation as $X_{i}=Q\left(k\right){\tilde{X}}_{i}$, where $X_{i}≜{\left[X_{i}^{0}\left(k\right),\cdots \;,X_{i}^{t}\left(k\right),\cdots \;,X_{i}^{N_{t}-1}\left(k\right)\right]}^{T}$ denotes precoding process output. $X_{i}={\tilde{X}}_{i}$ where *Q*(*k*) is an identity matrix.

At the *r*th receiver, the *i*th received OFDM symbol in time domain can be designated as $y_{i}^{r}\in {\mathbb{C}}^{\left(N_{cp}+N_{sc}\right)}$ in Equation 2:


(2) $$\begin{array}{l} {\text{y}}_{\text{i}}^{\text{r}}≜{\left[{\text{y}}_{\text{i}}^{\text{r}}\left(0\right),\cdots \;,{\text{y}}_{\text{i}}^{\text{r}}\left(\text{n}\right),\cdots \;,{\text{y}}_{\text{i}}^{\text{r}}\left({\text{N}}_{\text{cp}}+{\text{N}}_{\text{sc}}-1\right)\right]}^{\text{T}} \end{array}$$

and its frequency domain can be denoted as Equation 3:


(3) $$\begin{array}{l} {\text{Y}}_{\text{i}}^{\text{r}}≜{\left[{\text{Y}}_{\text{i}}^{\text{r}}\left(0\right),\cdots \;,{\text{Y}}_{\text{i}}^{\text{r}}\left(\text{k}\right),\cdots \;,{\text{Y}}_{\text{i}}^{\text{r}}\left({\text{N}}_{\text{sc}}-1\right)\right]}^{\text{T}} \end{array}$$

Symbol detection is on the receiver side, recovering all transmitted data streams ${\tilde{\text{X}}}_{\text{i}}^{\text{t}}$ by processing simultaneously received signals ${\text{y}}_{\text{i}}^{\text{r}}$ from all receivers.

**Figure 2:**
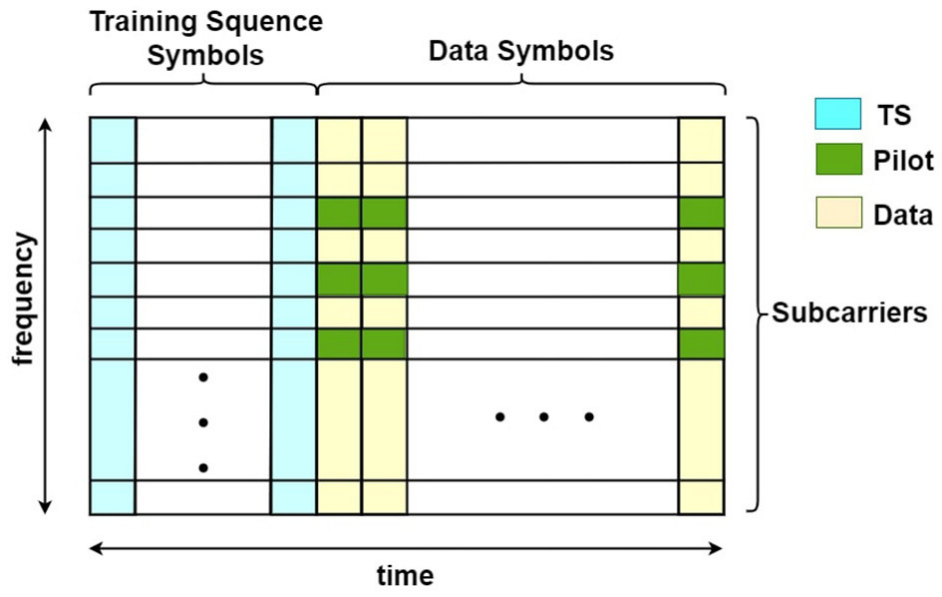
An overview of OFDM frame.

In Figure 2, we show the structure of a data frame with OFDM symbols. The first several symbols are designated as the training sequence (TS), which is usually for synchronization. The rest are data symbols containing the payload. Each OFDM symbol is divided into subcarriers in the frequency domain. In our simulation, subcarriers at fixed locations of the data symbols are used for model training, which are called pilots. With both TS symbols and pilots, we are able to get our AI model trained continuously in the data transmission.

### 2.2 Conventional method for mMIMO-OFDM symbol detection

Various methods exist in both the practical and theoretical domains for mMIMO-OFDM symbol detection. The most widely used method in practical application in mMIMO systems is LMMSE because of its low complexity among all. LMMSE, as a typical method based on channel estimation, is applied for mMIMO-OFDM symbol detection in the following two sequences:

**Step 1:** Estimation of the channel is based on the known TS symbols, which are designated by the first *N*_*TS*_ OFDM symbols in a frame. Then the TS symbols received on the RX side at *k*th subcarrier can be presented as Equation 4:


(4) $$\begin{array}{l} {\text{Y}}_{\text{TS}}\left(\text{k}\right)=\text{H}\left(\text{k}\right){\text{X}}_{\text{TS}}\left(\text{k}\right)+\text{G}, \end{array}$$

where *H*(*k*) is the channel response matrix and *X*_*TS*_(*k*) is the transmitted TS symbol matrix for the *k*th subcarrier. *G* indicates Gaussian noise matrix with variance σ^2^. Since the transmitted symbol matrix *X*_*TS*_(*k*) and the recovered symbol matrix *Y*_*TS*_(*k*) are already known, the channel model can be estimated using Equation 5:


(5) $$\begin{array}{l} Ĥ\left(k\right)=\frac{Y_{TS}\left(k\right)X_{TS}^{*}\left(k\right)}{X_{TS}\left(k\right)X_{TS}^{*}\left(k\right)+\sigma^{2}I}, \end{array}$$

Here [.^*^] means conjugate transpose matrix and Gaussian Noise matrix *G* = σ^2^*I* where *I* is an identity matrix.

**Step 2:** In this step, symbol detection is performed with the estimated channel model inherited from step 1, for the unknown symbols. If the *i*th transmitted symbol is ${\hat{X}}_{i}\left(k\right)$ and recovered symbol for the same sequence is Ŷ_*i*_(*k*) for the *k* sub-carrier, the recovered symbol sequence $\hat{Y_{i}\left(k\right)}$ can be represented as Equation 6:


(6) $$\begin{array}{l} {Ŷ}_{i}\left(k\right)=Ĥ\left(k\right){\hat{X}}_{i}\left(k\right)+G, \end{array}$$

So, transmitted *i*th symbol for k sub-carrier ${\hat{X}}_{i}\left(k\right)$ can be recovered using Equation 7:


(7) $$\begin{array}{l} {\hat{X}}_{k}\left(i\right)=\frac{{Ĥ}^{*}\left(k\right)Y_{i}\left(k\right)}{{Ĥ}^{*}\left(k\right)Ĥ\left(k\right)+\sigma^{2}I}, \end{array}$$

This is how the OFDM symbols can be recovered with the estimated channel model in the LMMSE method. Although this method is widely used in real-time applications due to its linearity and low complexity, it still suffers some limitations. The channel noise distribution has to be known beforehand. The channel response estimation must also be calculated accurately for the training sequence before starting to recover the unknown symbols. Such dependency on a pre-estimated model makes the performance limited with varying channel environments and with signals having a low signal-to-noise (SNR) ratio. Limited performance with challenging scenarios will be demonstrated in the result section.

In addition to LMMSE, there are some other well-known methods for such symbol detection tasks. However, they are not prioritized for practical situations for various reasons. Two of the well-known existing methods are as follows:

- **The maximum likelihood method:** This method operates by choosing the hypothesis that maximizes the likelihood function for the given received signal. It can theoretically provide the optimal solution. However, it has a major limitation due to its exponential complexity. For applications such as massive MIMO systems, the computational requirement grows exponentially with the size of the system, making such methods impractical to implement in real-time systems.
- **Sphere decoding (SD):** The SD method is an efficient non-convex solver that can provide highly optimized performance from methods like maximum likelihood symbol detection. Despite its effectiveness in a mathematical perspective, it becomes challenging to implement in real-time systems due to the high computational demand compared to linear receivers. Such computational demand becomes more complex in larger systems like massive MIMO systems where a larger number of receivers are required.

The ESN-based method is superior to the LMMSE model, too, because it does not require prior knowledge such as noise variance information of the channel. In the research from Shafin et al. (2018), ESN-based symbol detectors were also shown to be less energy-consuming for an LMMSE-based transmitter-receiver system. In our previous research from Zhou et al. (2020b), the ESN-based method for symbol detection of both SISO and MIMO has been found to surpass the limitations mentioned above from traditional methods in the aspect of computational complexity. The conclusion is reached under the condition of a large number of OFDM subcarriers, which also works for the massive MIMO systems.

### 2.3 Introduction to ESN

**Figure 3:**
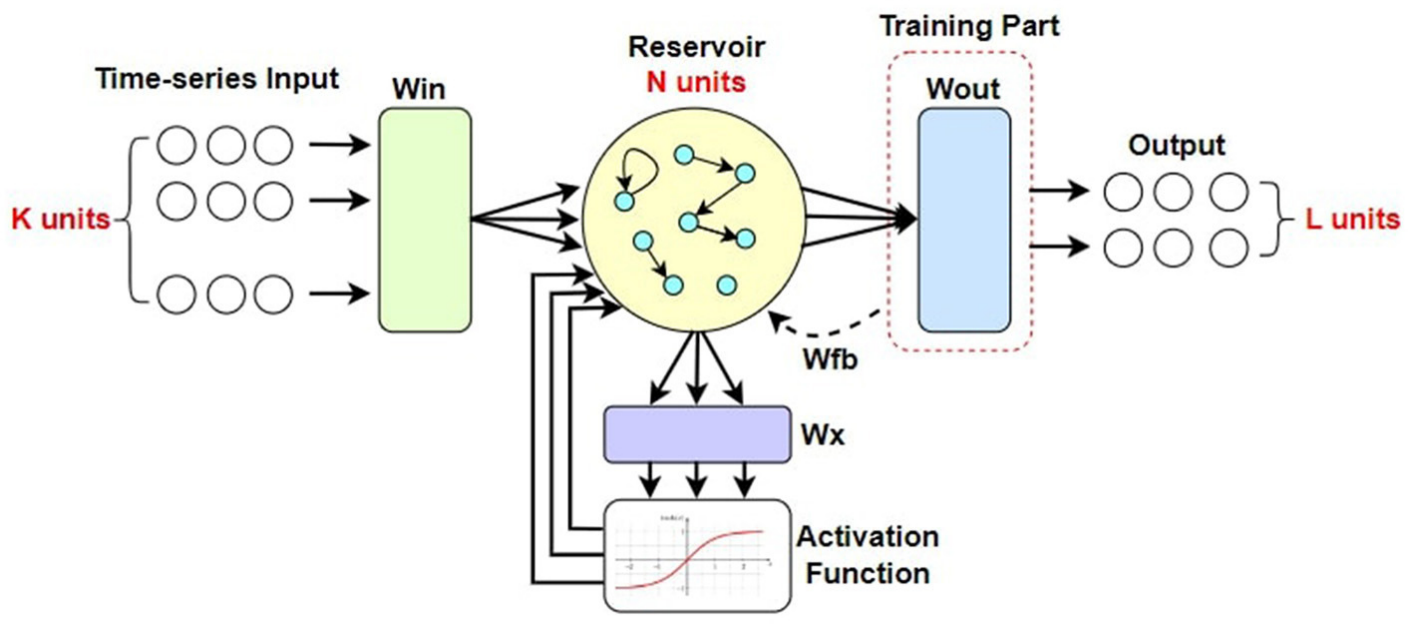
The architecture of a typical ESN.

ESN is a computationally efficient artificial neural network (ANN) that has shown its effectiveness in chaotic time-serial tasks. It was initially introduced by Jaeger and Haas (2004) who applied ESN to non-linear chaotic system prediction through supervised learning. A typical architecture of ESN model is provided in Figure 3.

ESN has even been shown to outperform recurrent neural networks **(RNN)** for temporal pattern detection and information processing tasks as learned from Jaeger (2001, 2002). ESN uses its reservoir to create a high-dimensional representation of its input features and provide the inherent dynamics (Gallicchio and Micheli, 2011; Lukoševičius et al., 2012). If the number of input neurons of an ESN model is *N*_*in*_ and the current input data is $x\left(n\right)\in {\mathbb{R}}^{N_{in}}$, along with *N*_*res*_ reservoir neurons producing state output $s\left(n\right)\in {\mathbb{R}}^{N_{res}}$, the concurrent state representation equation which governs the reservoir dynamics can be written as Equation 8:


(8) $$\begin{array}{l} \text{s}\left(n\right)=f\left(W^{in}x\left(n\right)+W^{res}s\left(n-1\right)+W^{fb}y\left(n-1\right)\right), \end{array}$$

Here, reservoir weight matrix is $W_{res}\in R^{N_{res}\times N_{res}}$ and input weight matrix is $W_{in}\in R^{N_{res}\times N_{in}}$ along with output feedback weight matrix as $W_{fb}\in R^{N_{res}\times N_{out}}$. For our system, we do not consider the feedback weight connection for simplification of the state equation. Since *W*^*fb*^ = 0, the equation becomes simplified to Equation 9:


(9) $$\begin{array}{l} \text{s}\left(n\right)=f\left(W^{in}x\left(n\right)+W^{res}s\left(n-1\right)\right), \end{array}$$

The extracted state *s*(*n*) of the reservoir tends to contain the most recent information and loses memory of past events gradually (Lukoševičius et al., 2012). This dynamic state is then fed into the output layer of *N*_*out*_ neurons which generates the final output *y*(*n*). *f*(.) is the non-linear activation function on the output of each reservoir neuron unit and is implemented element-wise. The reservoir state *s*(*n*) is initialized as a zero vector. The output *y*(*n*) can be represented by a combination of the reservoir state *s*(*n*) and the input *x*(*n*) as Equation 10:


(10) $$\begin{array}{l} \text{y}\left(n\right)=g\left({\left(W^{out}\right)}^{T}\left[s\left(n\right):x\left(n\right)\right]\right), \end{array}$$

where [*s*(*n*):*x*(*n*)] = *z*(*n*) is an extended system state and *g*(*n*) is the output activation function. The training for the ESN can be implemented in the following steps:

The original design of the ESN contains the following steps.

**Step 1:** Initialize the random reservoir and input weights *W*^*res*^, *W*^*in*^.

**Step 2:** All the input samples *N*^*train*^ are fed into the ESN as input *X* = {*x*(1), ..., *x*(*N*^*train*^)} and corresponding reservoir state vectors *z*(*n*)^*T*^ = [*s*(*n*):*x*(*n*)]^*T*^ are collected in a matrix $Z\in R^{N_{out}\times \left(N_{res}+N_{in}\right)}$ and the target labels are stored in a matrix $Y^{label}\in R^{\left(N_{out}\times 1\right)}$.

**Step 3:** We take the loss function for the ESN network regression prediction problem as MSE (Mean Square Error) and the ESN works on tuning the weights *W*^*out*^ to minimize MSE as much as possible. The MSE can be represented as Equation 11:


(11) $$\begin{array}{l} MSE=\min\|{\text{Y}}^{\text{predict}}-{\text{Y}}^{\text{label}}{\|}_{2}^{2} \end{array}$$

which can be rewritten to Equation 12,


(12) $$\begin{array}{l} MSE=\min\|\text{Z}{\text{W}}^{\text{out}}-{\text{Y}}^{\text{label}}{\|}_{2}^{2} \end{array}$$

Here, ||.||_2_ is denoted as the *l*_2_ norm. If the output activation function *g*(.) is an identity function then the *W*^*out*^ can be solved as least square estimation by taking the Moore-Penrose inversion of matrix *Z* using Equation 13,


(13) $$\begin{array}{l} {\hat{\text{W}}}_{\text{out}}={\text{Z}}^{†}{\text{Y}}^{\text{label}} \end{array}$$

where Ŵ_out_ is the estimation of *W*_out_.

**Step 4:** After getting the output weights trained, the ESN network can be inferred upon new testing data samples *N*^*test*^ so that the predicted output *y*(*n*) = {*y*(1), *y*(2)...*y*(*N*^*test*^)} can be computed using the estimated weights Ŵ_out_.

### 2.4 ESN-based symbol detector for massive MIMO

**Figure 4:**
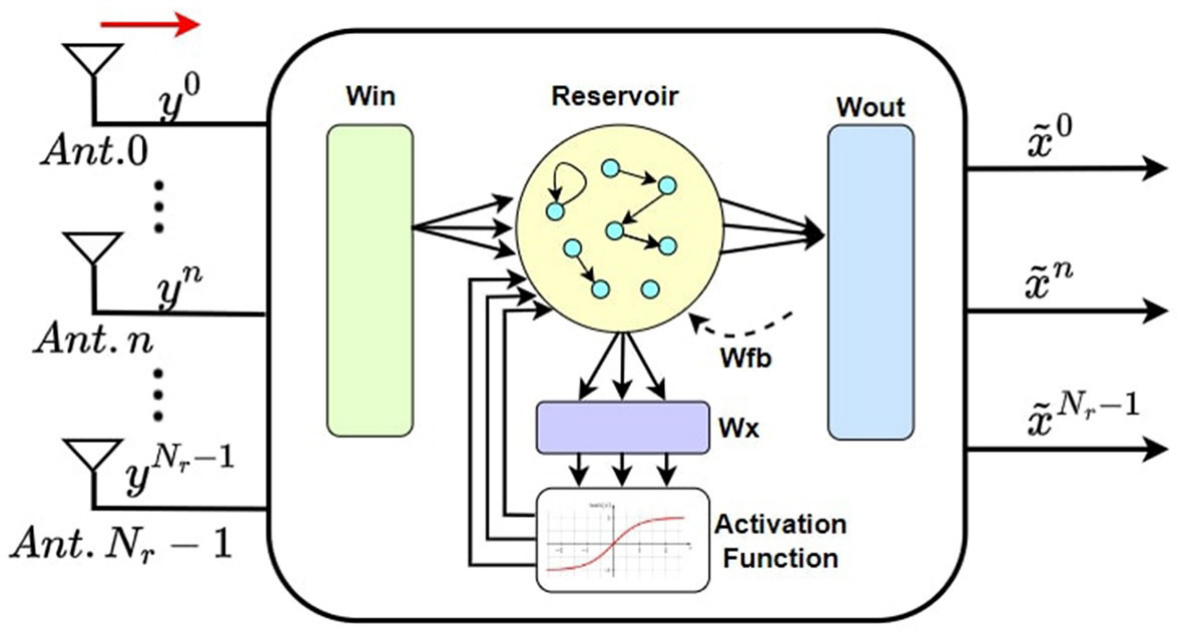
ESN-based symbol detection at Rx of a massive MIMO system.

A brief overview of the architecture of the ESN detector is provided in Figure 4. The recovered symbols at the RX antenna are denoted as $y_{i}^{r}$, which are then sent to the ESN detector. At the output of ESN, the predicted transmitted symbols $\hat{x_{i}^{t}}$ are generated. Inside the ESN detector, the training methodology is carried out in two steps.

**TS training:** Train the ESN with the *N*_*TS*_ pre-known TS symbols for which the target labels are already known. The training tuple on this step is created as Equation 14.


(14) $$\begin{array}{l} \text{Tuple}\left(Input_{TS};Label_{TS}\right)=\left\{y_{1},y_{2},\ldots ,y_{NTS};x_{1},x_{2},\ldots ,x_{NTS}\right\} \end{array}$$

Here, $x_{i}^{t}=$ [*x*_1_, *x*_2_, …, *x*_*NTS*_] represents the target label of the TS symbols. The output weights *W*^*out*^ of the ESN reservoir are trained for the above tuple using the Equation 13.

**Pilot training:** After training with the TS symbols, for each *i*th symbol where *i* > *N*_*TS*_, the pilot training tuple is prepared. The input and target label for the pilot symbol sequence can be described as Equations 15 and 16:


(15) $$\begin{array}{l} \text{I}nput_{pilot,i}\equiv {\left(Input_{pilot,i}^{0},\ldots ,Input_{pilot,i}^{N_{r}-1}\right)}^{T} \end{array}$$

(16) $$\begin{array}{l} \text{L}abel_{pilot,i}\equiv {\left(Label_{pilot,i}^{0},\ldots ,Label_{pilot,i}^{N_{t}-1}\right)}^{T} \end{array}$$

In order to prepare pilot training input $Input_{pilot,i}^{r}$ the data subcarriers of the received frame $Y_{i}^{r}$ are nulled, and then it is converted to time domain where CP is added with the time domain sequence using Equation 17:


(17) $$\begin{array}{l} \text{I}nput_{pilot,i}^{r}=Add_{cp}\left(F^{H}\Sigma Y_{i}^{r}\right) \end{array}$$

*F*^*H*^ signifies inverse Fourier transform matrix; Σ denotes diagonal matrix where the entries are 0 in data sub-carrier positions and 1 in pilot positions diagonally. The training labels for pilot symbols are also prepared in a similar way using Equation 18:


(18) $$\begin{array}{l} \text{L}abel_{pilot,i}^{t}=Add_{cp}\left(F^{H}\Sigma X_{i}^{t}\right) \end{array}$$

After training the ESN with the prepared pilot tuples, the output weights are updated and ESN takes the next *i*th symbol at receiver *y*_*i*_ and infers the transmitted symbol $\hat{x_{i}}$. This type of method is called the recursive method, which is more efficient for training ESN weights than the one-shot matrix inversion method since the ESN learning parameters are updated on a timely basis with incoming new training pilot samples. This helps the ESN to track the changing environment of the channel and provide better accuracy.

## 3 Reconfigurable ESN architecture design

### 3.1 Introduction to DSP48E1 IP

To design a high processing speed and power-efficient architecture, DSP48E1, a dedicated DSP IP block provided by Xilinx, was exploited in the design. As designed for high-speed signal processing tasks, it can perform arithmetic operations (i.e., multiplications, additions, subtractions, and accumulations) efficiently. Considering the large amount of multiplication and accumulation (MAC) operations in ESN implementation, DSP48E1 slices can play a significant role in the processing speed, accuracy, and power consumption of the accelerator.

The use of DSP48E1 can cut down the utilization of combinational logic blocks **(CLB)** which can take significantly higher space with low processing power. Generally, CLB-built multipliers are avoided in digital circuits to make the design cost-efficient. But this assumption is not true for DSP-built designs that have vendor-provided optimized circuit design. The approximate computing architecture of the **DSP** (Digital Signal Processor) units using efficient algorithms can perform multiplication efficiently with less power and with high speed (Immareddy and Sundaramoorthy, 2022). DSP IP slices are provided in almost all 7-series boards such as Xilinx Artix-7, Kintex-7, Virtex-7 and Zynq-7000. Virtex-7 series FPGA has 2800 DSP slices, which is significantly higher compared to other similar generation boards (Gan et al., 2021).

**Figure 5:**
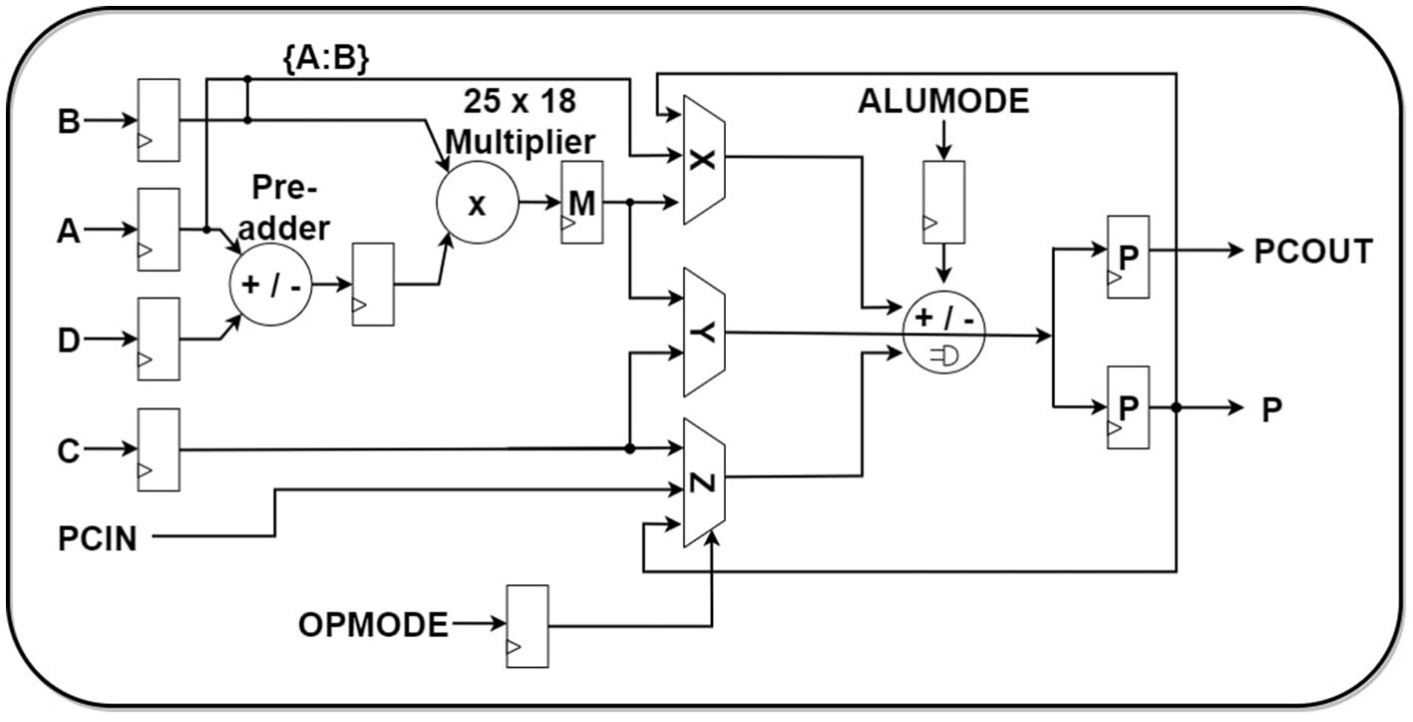
DSP48E1 IP overview.

From Figure 5, the short overview of DSP48E1 architecture can be seen. Significant blocks of the DSP slice that will be relevant to our architecture configuration are: (1) a 25 × 18 optimized two's complement binary multiplier, (2) a pattern detector, (3) a 48-bit accumulator, and (4) a low power pre-adder. The inputs to the DSP slice can be stored in the four input registers named **A,B,C,D**, and the output can be stored in the **P** register. The combination logic between the output P and the input registers can be shown by the Equation 19:


(19) $$\begin{array}{l} \text{P}=\text{C}\pm \left(\text{B}\times \left(\text{A}\pm \text{D}\right)\right) \end{array}$$

The inputs to the A and B registers can be concatenated up to 48 bits **(i.e., [A:B])** through a design provision and then can be used as one of the inputs to the 3-input SIMD ALU. Another feature that can play a significant role in compressor operations is the exploitation of PCIN/PCOUT ports as interconnections among DSP slices. This can help in parallel operation execution through a pipe-lining facility. These pipeline facility ports are only exploitable for DSP interconnections. The sophisticated architecture designed in this paper takes advantage of this feature which helps to improve its performance for Multiplication and Accumulation operation **(MACC)** against the custom CLB-based binary multipliers and adders significantly.

### 3.2 ESN configuration of DSP48E1 IP

The ESN reservoir synapses generate the state representation by getting input data from the input neurons and also previous states from the state memory. From Equation (9), it can be summarized that the related mathematical operation for the ESN state generation can be done in two sequential stages: (1) Calculate the linear combination of vector-matrix multiplication of the weight matrix {*W*^*res*^; *W*^*in*^} and data vectors {*s*(*n* − 1);*x*(*n*)} and (2) Applying a non-linear function operation *f*(.) on the result from the previous step. In this section, we explain the hardware configuration in FPGA for the 2 × 2 MIMO system OFDM symbol detection as a proof of concept.

In our design, each neuron inside the reservoir has to perform (*N*_*res*_ + *N*_*in*_) number of multiplications and (*N*_*res*_ + *N*_*in*_ − 1) number of additions at each time step. For the 2 × 2 MIMO-OFDM symbol detection, our sophisticated configuration was able to perform all the (8+40) multiplications and (8+40 − 1) number of adders with only nine DSPs demanded by each neuron. We tried to avoid the traditional approaches like using (*N*_*res*_ + *N*_*in*_) DSP slices for multiplication and $log_{2}^{\left(N_{res}+N_{in}\right)}$ compressor tree adders. Such an approach would make the design less efficient since there are so many CLB-based adders, which results in less power efficiency and more processing time demand (Xilinx, 2018) and also introduces difficulty in scalability when a large neural network is built with neurons of similar design.

In addition, our configuration was able to achieve both stages of calculation from matrix-vector multiplication and non-linear function approximation by using the same nine DSPs. The configuration was designed to use the pipelining capacity of the DSP slices and achieve parallel execution, which almost eliminates the need for CLBs for the execution of Equation (9) by any reservoir neuron.

**Figure 6:**
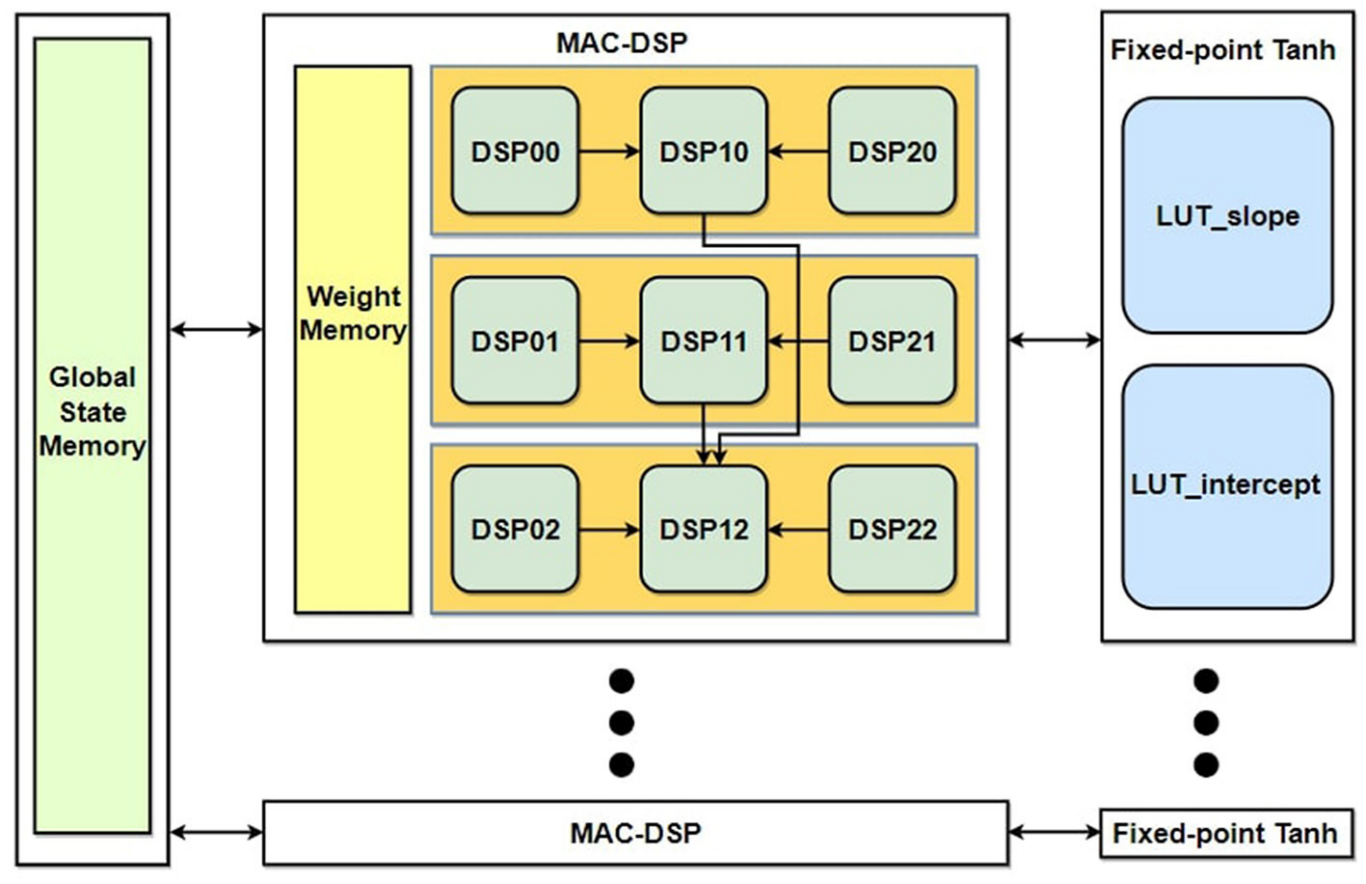
Proposed reservoir neuron architecture.

The architecture of a single reservoir neuron is shown in Figure 6. The weight parameters of a single neuron from both *W*^*in*^ and *W*^*res*^ are saved in the local weight memory register. The configuration formation of the nine DSPs that are required to complete the full operation of Equation (9) can be divided into four different stages, where the first three stages are used for the linear combination operation and the last stage is used for the application of hyperbolic tangent functions.

A basic pseudo-code listing of the different configurations of DSP slices for the first three stages used for the vector-matrix multiplication is written in Algorithm 1. The DSP_ij corresponds to the sequence of DSPs labeled in Equation (6) for each reservoir neuron, and the input/output ports labels can be associated with Equation (5). **Note:** Some of the statements are executed parallelly in the hardware DSP, which will be explained in detail in the following paragraph.

- Stage I: In the first stage, all DSPs of the neuron will load, multiply, and accumulate nine groups of weights and states. The ALUMODE and OPMODE of Figure 5 are configured to perform Equation 20:
  (20) $$\begin{array}{l} \text{P}=\text{P}\pm \left(\text{B}\times \text{A}\right) \end{array}$$
- Stage II: At this stage, the PCIN/PCout ports of the DSP slices are used for pipeline application. Every group of three DSPs **(i.e., DSP_0j, DSP_1j, DSP_2j)** is joined using the pipeline. Two DSPs (DSP_0j & DSP_2j) retain their SoPs in their P registers inherited from the first stage by setting up their own input registers A & B as 0. The ALUMODE & OPMODE configuration of DSP_1j is set up to perform Equation 21:
  (21) $$\begin{array}{l} \text{P}=\text{PCIN}+\text{C}+\text{P}, \end{array}$$
  The stage takes 2 clock cycles to perform all the required operations as shown in the pseudocode Algorithm 1. Every group of three SoPs are compressed into one SoP at the end of this stage.
- Stage III: At the end of the second stage operation, only three DSPs' output register P (i.e., DSP_10, DSP_11 & DSP_12) out of the nine DSPs retain data, where each DSP contains compressed SoP gained from second stage input of three SoPs. In the third stage, two of these DSPs' (DSP_10, DSP_11) *P*-register values are sent as input to the third DSP (DSP_12) which already contains one SoP in its own P register. All three SoPs are accumulated into one SoP using the three-input SIMD ALU which is configured to perform the operation of Equation 22 in DSP_12:
  (22) $$\begin{array}{l} \text{P}=\left\{A:B\right\}+\text{C}+\text{P}, \end{array}$$

**Algorithm 1:**
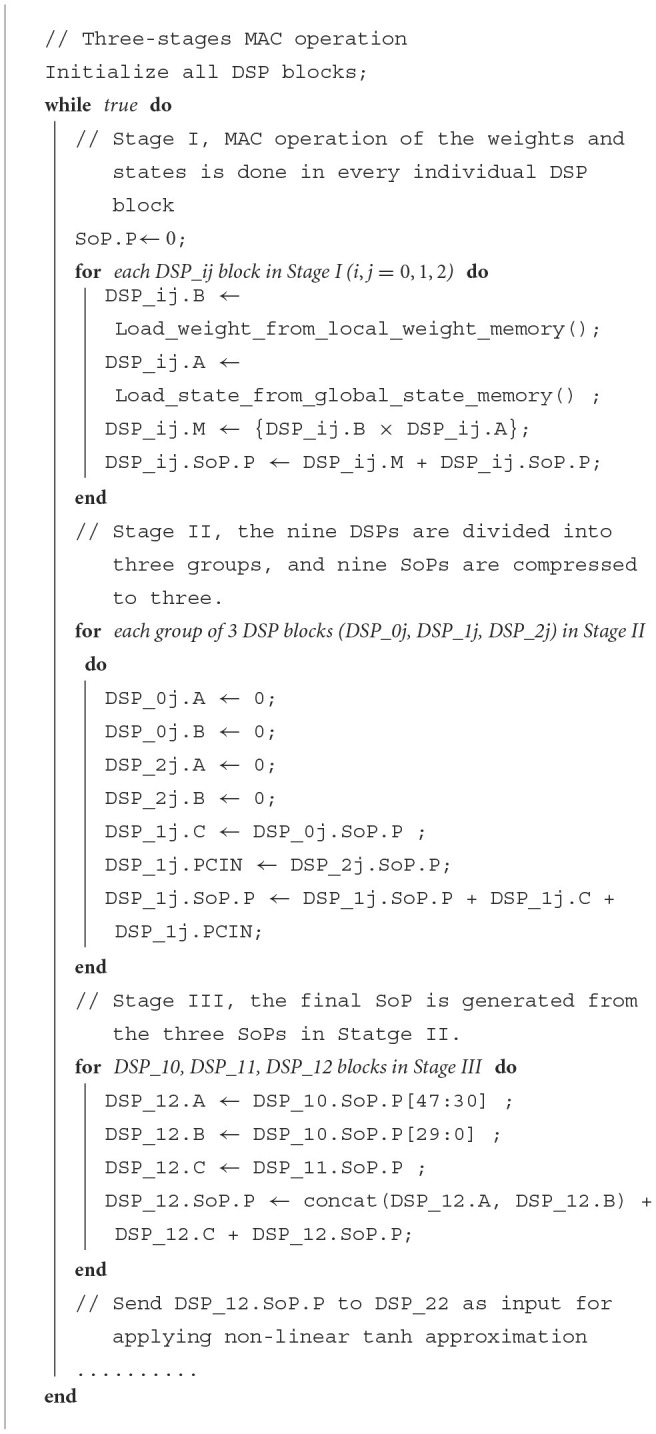
Pseudo code for the three-stage MAC operation. The DSP notation matches (Figure 6).

For each reservoir neuron, it needs to process (*N*_*res*_ + *N*_*in*_) number of weights and states in total at each epoch. For all nine DSPs of each neuron, it takes (⌈(*N*_*res*_ + *N*_*in*_)/9⌉ + 1) **(at Stage I)** + 2 **(at Stage II)** + 2 **(at Stage III)** clock cycles to process all these weights & states and complete the Matrix-Vector multiplication operation of the ESN reservoir in each epoch.

**Figure 7:**
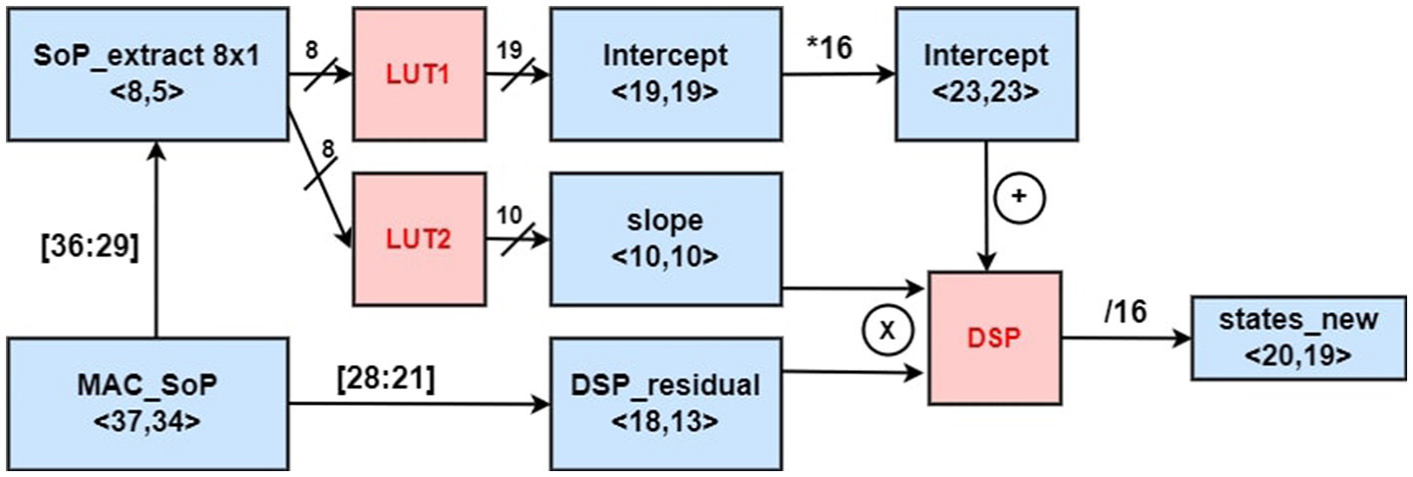
Non-linear fixed point approximation.

Next, the resultant SoP of stage III is sent to the DSP_22 for hyperbolic-tangent approximation. For efficient area utilization in the hardware, this non-linear application was designed taking a fixed-point approach. A brief overview of the design can be noticed in Figure 7. Here, we use the notation of < *l, f* > for a fixed-point number design with a total bitwidth of *l* and *f* fractional bits. The fixed point design exploits two LUTs (lookup tables). The contents of LUTs are used for estimating a slope and an intercept. The LUTs are generated using a similar method learned from Bajger and Omondi (2008). To apply this method, the tanh function is simplified using first-order piece-wise linear approximation in the Equation 23:


(23) $$\begin{array}{l} \tanh\left(x\right)\approx \{\begin{array}{cc} -1+error & \text{for}x\leq -a\\ slope\times x+intercept & \text{for}-a<x<a\\ 1-error & \text{for}x\geq a \end{array}, \end{array}$$

Since the tanh is a symmetrical function, just estimating the function output for the positive half of the input using LUTs can be sufficient. Moreover, when |*x*| ≥ 8, tanh output tends to be almost 1 with *error* ≡ 0 (<10^−6^). Therefore, we take *a* = 8 in our design.

The resulting SoP of the vector-matrix operation is fed as an index input to both LUTs. After extracting the slope and intercept values from two individual LUTs separately, they are fed into another DSP unit to perform the MAC operation on the equation (*slope* × *x* + *intercept*).

Considering that the limitation bit width of the DSP multiplier (25 × 18) and the importance of utilization control, the input to both lookup tables are taken by truncating the MSB and redundant LSBs *LUT*_*SoP*_ = |*SoP*|_36:29_ which keeps the error of tanh output within the range of 10^−6^. Since the input of the LUT is 8-bit wide, the depth of both LUTs becomes 2^8^. The input *x* = |*SoP*|_28:21_ is also truncated, which is sufficient to generate results with good precision.

The final implementation of the ESN on the FPGA has 40 input neurons and four output neurons. The number of reservoir neurons was chosen to be 8, which was found to be the second-best parameter for producing accuracy in neurons. Parameter 8 was chosen to save hardware space as this halved the number of required DSPs in the ESN accelerator.

For the calculation of Equation 10, the width of the values in *W*^*out*^ also needed optimization. In our design, it was decided that *W*^*out*^ can be within the range of (−700, 700) for a normalized input range of (−0.1, 0.1). *W*^*out*^ was decided to be 16-bit wide with a precision of 2^−5^. A short table description of the parameter for the non-linear part and ESN structure for the proof of concept 2 × 2 MIMO is displayed in Table 1 for clarity.

**Table 1:** Parameter description of the ESN accelerator.

**Parameter**	**Value**
Input neurons	40
Reservoir neurons	8
Output neurons	4
Input range	(−0.1, 0.1)
Output range	(−700, 700)
a (in Tanh approximation)	8
Depth of Tanh LUTs	2^8^

## 4 Simulation results and analysis

**Figure 8:**
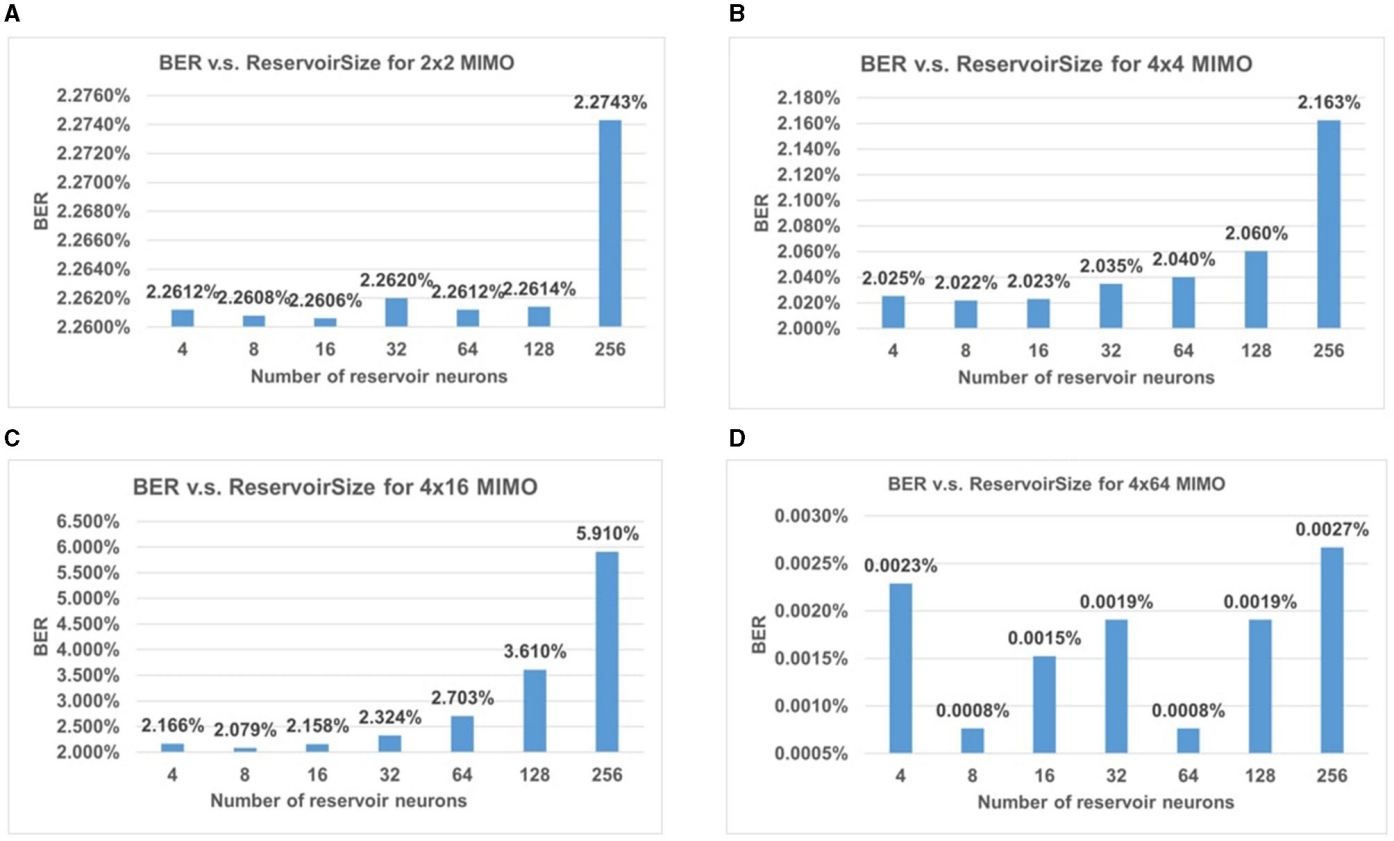
BER performance for different sizes of MIMO with changing reservoir size. The impact of reservoir size on BER performance for **(A)** 2 × 2 MIMO, **(B)** 4 × 4 MIMO, **(C)** 4 × 16 MIMO, and **(D)** 4 × 64 MIMO.

In conventional neural networks, the selection of parameters can significantly influence the accuracy of the proposed model. We conducted an experiment on the impact of reservoir size on BER performance for different settings of MIMO systems, including 2 × 2, 4 × 4, 4 × 16, and 4 × 64. Here we focus on the massive antennas at the receiver side in uplink transmission. The window length remains constant during the entire simulation. The results are shown in Figure 8. In each chart, the BER of the MIMO system is compared between different numbers of reservoir neurons, from 4 to 512. It shows that ESN with 8–16 neurons can achieve the lowest BER in most of the cases listed here.

We also compared the performance of the traditional LMMSE method with the ESN methods in different MIMO configurations. The number of OFDM subcarriers for each case is set to 1,024, which is a common value used in massive MIMO systems to support high data transmission. The BER comparison results in Table 2 reveal that the ESN symbol detector exceeds the LMMSE methods in all cases, showing its better recovery capability for the received OFDM symbols. And from the small MIMO system (2 × 2) to the larger MIMO system (4 × 64), the wireless channel becomes more complicated to model for these symbol detectors. LMMSE-based symbol detector fails to keep a good performance with increasing system size due to the limitation of its estimation capability. However, ESN is able to get trained with the signals and keep the model updated continuously. The BER of the ESN symbol detector is kept in a relatively low level and even achieves great results in 4 × 64 MIMO simulation.

**Table 2:** Comparison of the BER between ESN method and LMMSE method in different MIMO configurations.

**MIMO configuration**	**2 × 2**	**4 × 4**	**4 × 16**	**4 × 64**
BER of LMMSE method	5.460%	13.088%	3.167%	6.68o%
BER of ESN method	2.261%	2.022%	2.079%	0.001%

## 5 Proof of concept of the FPGA design

### 5.1 Hardware setup

A real-time hardware experiment was performed for 2 × 2 proof-of-concept MIMO-OFDM symbol detection on a software-defined radio(SDR) / FPGA joined testbed, which consists of a Xilinx Virtex-7 FPGA board, a GNU Radio software, and two universal software radio peripheral devices (USRPs).

**Figure 9:**
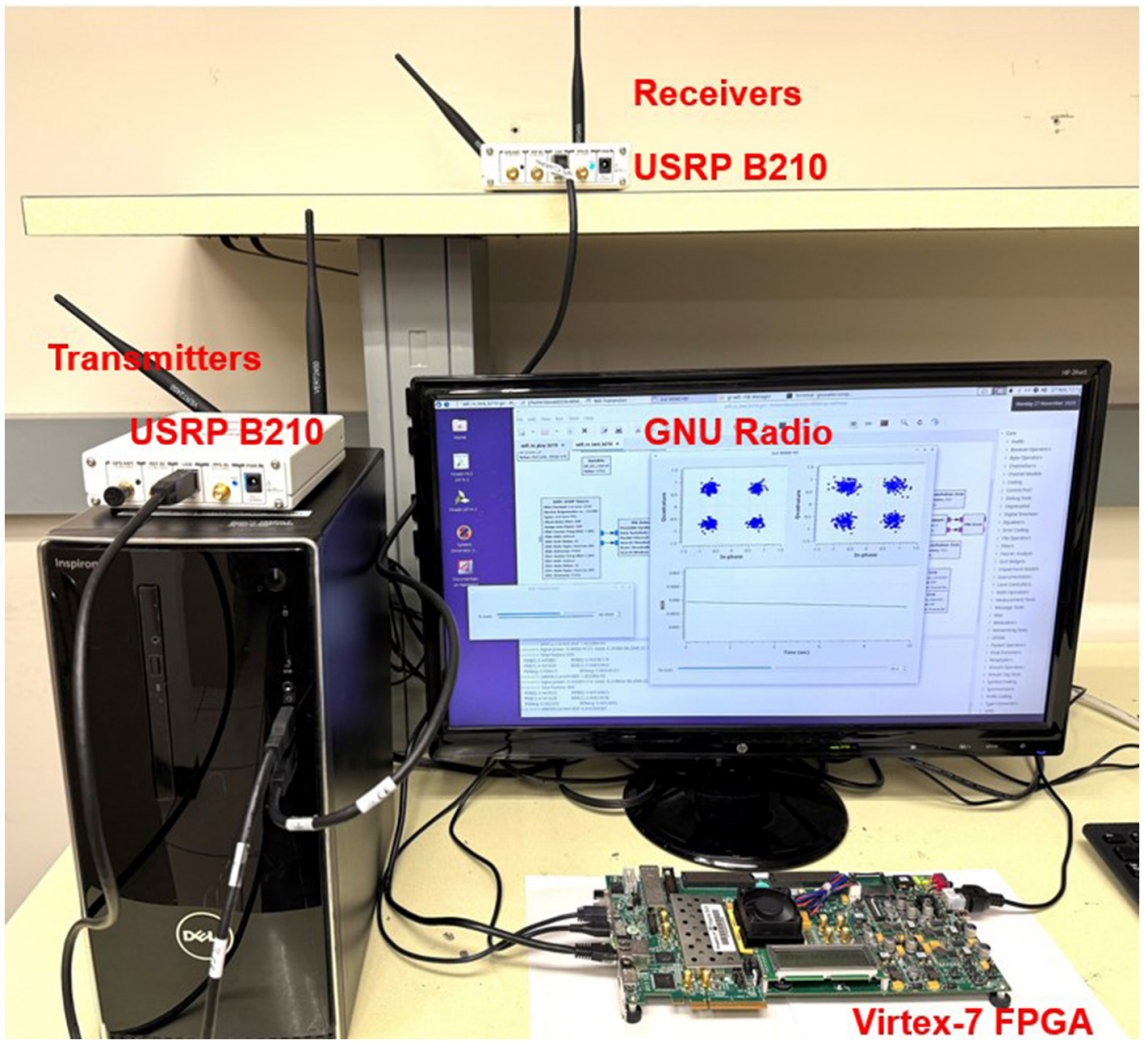
Setup of the SDR/FPGA jointed testbed.

In Figure 9, a MIMO-OFDM radio system was implemented using GNU Radio development software, where USRPs were used to transmit and receive RF signals. The received signal was sent to the ESN symbol detector on FPGA via a high-speed wired ethernet transmission. The ESN then performs the symbol detection task and sends back the predicted results for the transmitted signals to the GNU Radio application, where the accuracy is measured. Moreover, the LMMSE-based symbol detection is also performed in GNU Radio for comparison.

The design was validated in our RF lab where various wireless communication experiments were tested in real-world scenarios (Liang et al., 2022). Multipath scattering effects were presented in such environments. Various test scenarios were created using metal shelves and toolboxes to block the line of sight (LoS) between the TX/RX antennas. We performed FPGA testing in five different scenarios where each test was run for three trials without any change in antenna orientation or TX/RX position. The antenna orientation and positions of the transceivers are changed between different test scenarios to assess the prototype under different RF front-end gains. An overview of the setup of the five scenarios is given in Table 3.

**Table 3:** Setup of five test scenarios.

**Scenario**	**Description**
Scenario 1	Setting up Tx-Rx 10m apart (long distance) with clear line-of-sight (LoS) signal path.
Scenario 2	Setting up Tx-Rx 5m apart with non-line-of-sight signal path (NLoS) where the Tx & Rx are blocked partially.
Scenario 3	Tx-Rx placed further apart (NLoS) where Tx & Rx are fully blocked.
Scenario 4	Tx-Rx placed further apart (NLoS) and partially blocked.
Scenario 5	Tx-Rx placed furthest apart where the Tx/Rx are totally blocked.

### 5.2 FPGA synthesis results

In the FPGA onboard verification, we ran the real-time operation with a central clock speed of 125 MHz. From the synthesis report, the ESN architecture showed a dynamic power of 0.256 W and a static power of 0.262 W. The temperature report proves that the junction temperature remains around 25.9°C, which suffices to the board's requirement that the junction temperature should stay below 85°C, according to the Vivado temperature report.

The ESN implementation for 2 × 2 MIMO on the Virtex-707 board achieves almost 3.3 times the processing speed compared to a SISO-specific ESN implementation where the authors achieved 10.53 million input samples/s (Gan et al., 2021) for their FPGA implementation. Our processing speed reaches up to 34.8 million input sample/s. The proposed accelerator was able to save 50% BRAM memory usage and 33.3% DSP IP blocks compared to the SISO FPGA design above. Despite gaining such a high throughput, the design only increased the usage of LUT by 21.4% and FF by 33%, respectively. The detailed comparison of resource utilization between the two FPGA-based ESN implementation is shown in Table 4.

**Table 4:** Summary of resource utilization.

**Optimization type**	**Max processing speed**
LUT	13,314 (4.9%)
FF	10,750 (1.77%)
BRAM	6 (0.58%)
DSP	108 (3.86%)

## 6 Conclusions

In this research, we conducted significant efforts into AI-enabled 6G tasks with FPGA acceleration. The performance of ESN-based symbol detectors is experimented across MIMO systems with different configurations. The findings demonstrate the efficiency of the ESN architecture in handling various sizes of the MIMO system without substantial increases in reservoir size. Another key aspect of our work is to leverage the DSP slices within the ESN reservoir neuron architecture, significantly enhancing the cost-efficiency of the FPGA accelerator of ESN. Furthermore, our FPGA accelerator was validated through a proof-of-concept experiment. This not only affirmed the accuracy of our approach but also indicated better resource utilization than previous studies. However, because of the limitations of the equipment, we are not able to perform FPGA validation for the MIMO systems with a large number of antennas. And the potential of FPGA acceleration in massive MIMO can be explored even further.

## Data availability statement

The raw data supporting the conclusions of this article will be made available by the authors, without undue reservation.

## Author contributions

CL: Writing – original draft, Writing – review & editing. MA: Writing – original draft, Writing – review & editing. YL: Writing – review & editing. YY: Writing – review & editing.

## References

[B1] Bajger M. Omondi A. (2008). Low-error, high-speed approximation of the sigmoid function for large FPGA implementations. J. Signal Process. Syst. 52, 137–151. 10.1007/s11265-007-0140-z

[B2] Gallicchio C. Micheli A. (2011). Architectural and markovian factors of echo state networks. Neural Netw. 24, 440–456. 10.1016/j.neunet.2011.02.002 21376531

[B3] Gan V. M. Liang Y. Li L. Liu L. Yi Y. (2021). A cost-efficient digital ESN architecture on FPGA for OFDM symbol detection. ACM J. Emerg. Technol. Comp. Syst. 17, 1–15. 10.1145/3440017

[B4] He H. Wen C.-K. Jin S. Li G. Y. (2018). Deep learning-based channel estimation for beamspace mmwave massive MIMO systems. IEEE Wireless Commun. Lett. 7, 852–855. 10.1109/LWC.2018.283212835632347

[B5] Hoydis J. Aoudia F. A. Valcarce A. Viswanathan H. (2021). Toward a 6G AI-native air interface. IEEE Commun. Mag. 59, 76–81. 10.1109/MCOM.001.2001187

[B6] Immareddy S. Sundaramoorthy A. (2022). A survey paper on design and implementation of multipliers for digital system applications. Artif. Intell. Rev. 55, 4575–4603. 10.1007/s10462-021-10113-0

[B7] Jaeger H. (2001). The “Echo State” Approach to Analysing and Training Recurrent Neural Networks-With an Erratum Note. Bonn: German National Research Center for Information Technology GMD Technical Report 148, 13.

[B8] Jaeger H. (2002). Adaptive nonlinear system identification with echo state networks. Adv. Neural Inf. Process. Syst. 15, 609–616.

[B9] Jaeger H. Haas H. (2004). Harnessing nonlinearity: predicting chaotic systems and saving energy in wireless communication. Science 304, 78–80. 10.1126/science.1091277 15064413

[B10] Liang Y. Li L. Yi Y. Liu L. (2022). Real-time machine learning for symbol detection in MIMO-OFDM systems, in IEEE INFOCOM 2022-IEEE Conference on Computer Communications (London: IEEE), 2068–2077.

[B11] Lin C. Liang Y. Yi Y. (2022). FPGA-based reservoir computing with optimized reservoir node architecture, in 2022 23rd International Symposium on Quality Electronic Design (ISQED) (Santa Clara, CA: IEEE), 1–6.

[B12] Liu S. Wang T. Wang S. (2021). Toward intelligent wireless communications: Deep learning-based physical layer technologies. Digit. Commun. Netw. 7, 589–597. 10.1016/j.dcan.2021.09.014

[B13] Lukoševičius M. Jaeger H. Schrauwen B. (2012). Reservoir computing trends. Künstliche Intelligenz 26, 365–371. 10.1007/s13218-012-0204-5

[B14] Marzetta T. L. (2010). Noncooperative cellular wireless with unlimited numbers of base station antennas. IEEE Transact. Wireless Commun. 9, 3590–3600. 10.1109/TWC.2010.092810.091092

[B15] Mosleh S. S. Liu L. Sahin C. Zheng Y. R. Yi Y. (2017). Brain-inspired wireless communications: where reservoir computing meets MIMO-OFDM. IEEE Transact. Neural Netw. Learn. Syst. 29, 4694–4708. 10.1109/TNNLS.2017.2766162 29990240

[B16] Neumann D. Joham M. Utschick W. (2015). Channel estimation in massive MIMO systems. arXiv [preprint]. 10.48550/arXiv.1503.08691

[B17] Qin Z. Ye H. Li G. Y. Juang B.-H. F. (2019). Deep learning in physical layer communications. IEEE Wireless Communications 26, 93–99. 10.1109/MWC.2019.1800601

[B18] Rusek F. Persson D. Lau B. K. Larsson E. G. Marzetta T. L. Edfors O.. (2012). Scaling up mimo: Opportunities and challenges with very large arrays. IEEE Signal Process. Mag. 30, 40–60. 10.1109/MSP.2011.2178495

[B19] Saad W. Bennis M. Chen M. (2019). A vision of 6g wireless systems: applications, trends, technologies, and open research problems. IEEE Netw. 34, 134–142. 10.1109/MNET.001.1900287

[B20] Sah D. K. Kumar D. P. Shivalingagowda C. Jayasree P. (2019). 5g Applications and Architectures. 5G Enabled Secure Wireless Networks. Cham: Springer, 45–68.

[B21] Shafin R. Liu L. Ashdown J. Matyjas J. Medley M. Wysocki B.. (2018). Realizing green symbol detection via reservoir computing: an energy-efficiency perspective, in 2018 IEEE International Conference on Communications (ICC) (Kansas City, MO: IEEE), 1–6.

[B22] Sohrabi F. Attiah K. M. Yu W. (2021). Deep learning for distributed channel feedback and multiuser precoding in FDD massive MIMO. IEEE Transact. Wireless Commun. 20, 4044–4057. 10.1109/TWC.2021.3055202

[B23] Tuchler M. Koetter R. Singer A. C. (2002a). Turbo equalization: Principles and new results. IEEE Transact. Commun. 50, 754–767. 10.1109/TCOMM.2002.1006557

[B24] Tuchler M. Singer A. C. Koetter R. (2002b). Minimum mean squared error equalization using a priori information. IEEE Transact. Signal Process. 50, 673–683. 10.1109/78.984761

[B25] Wang T. Wen C.-K. Wang H. Gao F. Jiang T. Jin S. (2017). Deep learning for wireless physical layer: opportunities and challenges. China Commun. 14, 92–111. 10.1109/CC.2017.8233654

[B26] Wen C.-K. Shih W.-T. Jin S. (2018). Deep learning for massive MIMO CSI feedback. IEEE Wireless Commun. Lett. 7, 748–751. 10.1109/LWC.2018.2818160

[B27] Wu M. Yin B. Wang G. Dick C. Cavallaro J. R. Studer C. (2014). Large-scale MIMO detection for 3GPP LTE: Algorithms and FPGA implementations. IEEE J. Sel. Top. Signal Process. 8, 916–929. 10.1109/JSTSP.2014.2313021

[B28] Xie H. Gao F. Jin S. (2016). An overview of low-rank channel estimation for massive MIMO systems. IEEE Access 4, 7313–7321. 10.1109/ACCESS.2016.2623772

[B29] Xilinx (2018). 7 Series DSP48E1 Slice User Guide (UG479). San Jose, CA: Xilinx, Inc.

[B30] Zhou Z. Liu L. Chandrasekhar V. Zhang J. Yi Y. (2020a). Deep reservoir computing meets 5G MIMO-OFDM systems in symbol detection. Proc. AAAI Conf. Artif. Intell. 34, 1266–1273. 10.1609/aaai.v34i01.5481

[B31] Zhou Z. Liu L. Chang H.-H. (2020b). Learning for detection: MIMO-OFDM symbol detection through downlink pilots. IEEE Transact. Wireless Commun. 19, 3712–3726. 10.1109/TWC.2020.2976004

